# Line-Field Confocal Optical Coherence Tomography for the Diagnosis of Skin Carcinomas: Real-Life Data over Three Years

**DOI:** 10.3390/curroncol30100639

**Published:** 2023-09-28

**Authors:** Carolina Donelli, Mariano Suppa, Linda Tognetti, Jean Luc Perrot, Laura Calabrese, Javiera Pérez-Anker, Josep Malvehy, Pietro Rubegni, Elisa Cinotti

**Affiliations:** 1 Department of Medical, Surgical and Neurological Sciences, Dermatology Section, University of Siena, 53100 Siena, Italy; linda.tognetti@unisi.it (L.T.); laura.calabrese@unisi.it (L.C.); pietro.rubegni@gmail.com (P.R.); elisa.cinotti@unisi.it (E.C.); 2 Groupe d’Imagerie Cutanée Non Invasive (GICNI), Société Française de Dermatologie (SFD), 75008 Paris, France; dr.marianosuppa@gmail.com (M.S.); j.luc.perrot@chu-st-etienne.fr (J.L.P.); 3 Department of Dermatology, Hôpital Erasme, Université Libre de Bruxelles, 1050 Brussels, Belgium; 4 Department of Dermatology, University Hospital of St-Etienne, 42270 Saint-Etienne, France; 5 Melanoma Unit, Dermatology Department, Hospital Clínic de Barcelona, IDIBAPS, Universitat de Barcelona, 08001 Barcelona, Spain; javiperezanker@gmail.com (J.P.-A.); jmalvehy@gmail.com (J.M.); 6 CIBER de Enfermedades Raras, Instituto de Salud Carlos III, 08007 Barcelona, Spain

**Keywords:** LC-OCT, optical coherence tomography, basal cell carcinoma, squamous cell carcinoma, non-melanoma skin cancer, sensitivity, specificity, accuracy, imaging

## Abstract

Line-field confocal optical coherence tomography (LC-OCT) can help the clinical diagnosis of skin diseases. The present study aimed to evaluate the sensitivity, specificity, and diagnostic accuracy of LC-OCT for the diagnosis of the most frequent non-melanoma skin cancers (NMSCs), i.e., basal cell carcinoma (BCC) and squamous cell carcinoma (SCC). Comparing LC-OCT diagnostic performances with those of dermoscopy, histopathological examination was used as a gold standard. For every study endpoint, the diagnostic ability of LC-OCT revealed superiority over the dermoscopic examination. In particular, a significant increase in specificity was observed. Sensitivity, specificity, and diagnostic accuracy of dermoscopy and LC-OCT for the diagnosis of malignancy were, respectively, 0.97 (CI 0.94–0.99), 0.43 (CI 0.36–0.51), and 0.77 (CI 0.72–0.81) for dermoscopy and 0.99 (CI 0.97–1.00), 0.90 (CI 0.84–0.94), and 0.96 (CI 0.93–0.97) for LC-OCT. The positive predictive value (PPV) resulted in 0.74 (CI 0.69–0.78) for dermoscopy and 0.94 (CI 0.91–0.97) for LC-OCT, and the negative predictive value (NPV) was 0.89 (CI 0.81–0.95) for dermoscopy and 0.98 (CI 0.95–1.00) for LC-OCT. Finally, our real-life study showed a potentially important role of LC-OCT in the non-invasive diagnosis of NMSCs, especially BCC. The real-time imaging technique could spare unnecessary biopsies with an increased sensitivity, a much higher specificity, and better accuracy than clinical assessment with dermoscopy alone.

## 1. Introduction

Line-field confocal optical coherence tomography (LC-OCT) is a real-time imaging technique. By combining the advantages of reflectance confocal microscopy (RCM) and optical coherence tomography (OCT) in terms of penetration, spatial resolution, and image orientation, it overcomes their respective limits [1]. LC-OCT images are produced by calculating the time of flight and amplitude of light backscattered from the tissue microstructures at up to ~500 µm depth [2,3]. The device reaches an isotropic resolution of ~1 µm thanks to the use of a supercontinuum laser source as a broadband source providing cellular axial resolution and high numerical aperture objectives in each arm of the interferometer to achieve a high lateral resolution. The LC-OCT device is capable of acquiring vertical section images, horizontal section images, as well as three-dimensional (3D) images with cellular resolution. In addition to in-depth images, it provides a dermoscopic color surface image of the skin. This image is acquired in real-time and in parallel to LC-OCT images, and it guides the skin surface exploration. Images are acquired with a handheld probe placed in contact with the skin, using a drop of paraffin oil between the skin and the tip of the probe to prevent parasitic reflections. Since it has been shown that the device can be helpful in the clinical diagnosis of different neoplastic [4,5,6,7,8,9], inflammatory [10,11], and infectious [12,13,14,15,16] skin diseases, LC-OCT has recently been gaining attention. Particularly, it appeared to be very effective in the identification of basal cell carcinoma (BCC) [17,18,19]. LC-OCT can even differentiate histologic subtypes of BCC and monitor non-invasive treatments [20,21]. Moreover, this in vivo diagnostic technique has demonstrated its usefulness in differentiating actinic keratosis (AK) from squamous cell carcinoma (SCC) [22,23,24,25]. The relevance of LC-OCT for the diagnosis of skin tumors has been illustrated by numerous descriptive studies [26]. The aim of the present one was to evaluate in a real-life setting the sensitivity, specificity, accuracy, positive predictive value (PPV), and negative predictive value (NPV) of the optical imaging device compared to dermoscopy for the diagnosis of the most frequent non-melanoma skin cancers (NMSCs), including BCC and SCC.

## 2. Materials and Methods

A prospective observational, monocentric study was performed. We examined the database of the LC-OCT device (DeepLive, Damae, France) of the Dermatology Department of the University Hospital of Siena. Data deidentification was performed before use, and the study was conducted as stated by the Declaration of Helsinki. It included all cutaneous lesions observed from 1 September 2020 to 3 February 2023 in the University Hospital of Siena with uncertain clinical and dermoscopic diagnoses of possible malignant skin tumors. Lesions on the eyelid margin, internal cantus of the eye, and upper eyelid were excluded because LC-OCT could damage the retina.

The LC-OCT examination was an adjunctive diagnostic tool that helped the decision of excising or following up on a lesion. Dermoscopic and LC-OCT diagnoses were prospectively entered into the LC-OCT software during the examination. The software had three fields named, respectively, “dermoscopic diagnosis”, “LC-OCT diagnosis”, and “patient management”, which should be filled for each lesion during the examination and which were taken into account for the present analysis. LC-OCT images and dermoscopic images were acquired by an expert in skin imaging, and diagnoses were given by a skin imaging expert dermatologist with more than 10 years of experience in dermoscopy and OCT (E.C.). Dermoscopic images were acquired with VivaCam D200 (VivaScope GmbH, Munich, Germany) at 15× magnification, and dermoscopic diagnoses were performed using both this videodermatoscope and the Dermlite DL200 Hybrid handheld dermatoscope (Dermlite, Aliso Viejo, CA, USA).

We analyzed only lesions suspicious of NMSCs (i.e., BCC, Bowen’s disease, and SCC) or their possible imitators, including “AK”, “intradermal nevus (IN)”, “seborrheic keratosis (SK)”, “sebaceous hyperplasia (SH)”, “inflammatory lesion (IL)”, and “other” at dermoscopic and/or LC-OCT examination. Melanocytic lesions (different from IN) and basosquamous cell carcinomas were excluded. In LC-OCT, the presence of tumor lobules was the main diagnostic feature of BCC [17]. The lesions suspicious for malignant skin tumors under dermoscopy were surgically removed (if confirmed by LC-OCT) or biopsied (if not confirmed by LC-OCT) for histological diagnosis. Lesions considered malignant at LC-OCT but not under dermoscopy were biopsied. Lesions considered benign at dermoscopy and/or LC-OCT were followed up for at least one year to exclude malignancy. Dermoscopic diagnoses also included clinical evaluation.

There were four study endpoints. Considering the group of lesions with histological diagnosis based on complete surgical excision, the first endpoint was to compare parameters such as sensitivity, specificity, accuracy, PPV, and NPV of dermoscopy versus those of LC-OCT in distinguishing BCC from its imitators (Bowen’s disease and SCC included). The second endpoint evaluated the same five parameters of the two non-invasive imaging techniques in terms of the ability to differentiate malignant (i.e., skin carcinomas including BCC, Bowen’s disease, and SCC and AKs) from benign (IN, SK, SH, IL, and “other”) lesions. AKs were considered to be in the malignant lesion group because they require treatment. We also evaluated the diagnostic ability of LC-OCT and dermoscopy for BCC (third endpoint) and malignant tumors (fourth endpoint) by analyzing both skin lesions that had a histological examination of a complete excision or skin biopsy and those that had a follow-up of more than one year. The exact 95% confidence interval (CI) was calculated for sensitivity, specificity, accuracy, PPV, and NPV of each endpoint.

## 3. Results

A total of 1481 skin lesions imaged with LC-OCT were analyzed. Three-hundred and sixty lesions suspicious of skin malignancy were surgically removed and had histopathologic diagnoses (Table 1).

In addition to the lesions that were not in differential diagnosis with skin cancers, we excluded 23 nevi, 15 melanomas, and 7 basosquamous cell carcinomas. We performed a sub-analysis of the remaining 312 lesions according to the different diagnoses such as BCC (152), AK (20), Bowen’s disease (14), SCC (22), IN (4), SK (14), SH (1), IL (15), and “other” (70). Then, 636 lesions with follow-up or skin biopsy were present in the database. Excluding the lesions that were not in differential diagnosis with skin cancers, 51 nevi, and 1 basosquamous cell carcinoma, 154 out of 636 lesions were selected. Therefore, a total of 466 lesions with both histopathology (312 completely excised and 149 biopsied) and follow-up (five cases) were analyzed (Table 2).

### 3.1. First Endpoint: BCC vs. Imitators Considering Completely Excised and Histopathologically Confirmed Lesions

**Figure 1:**
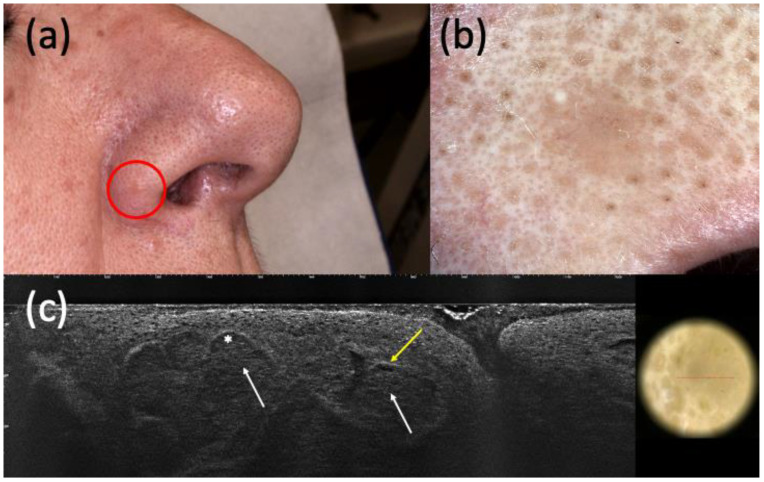
Histologically diagnosed nodular basal cell carcinoma (BCC) with non-specific clinical (**a**) and dermoscopic (**b**) examination but highly suspected in line-field confocal optical coherence tomography (LC-OCT) (**c**). It was a small pink papule on the right nasal ala (**a**) that showed a homogeneous pinkish pigmentation with some short fine telangiectasias (**b**) in dermoscopy. The presence of hypo-reflective roundish lobules (white arrows), clefting (white asterisk), and hyperreflective stroma (yellow arrow) in LC-OCT supported the hypothesis of BCC.

**Figure 2:**
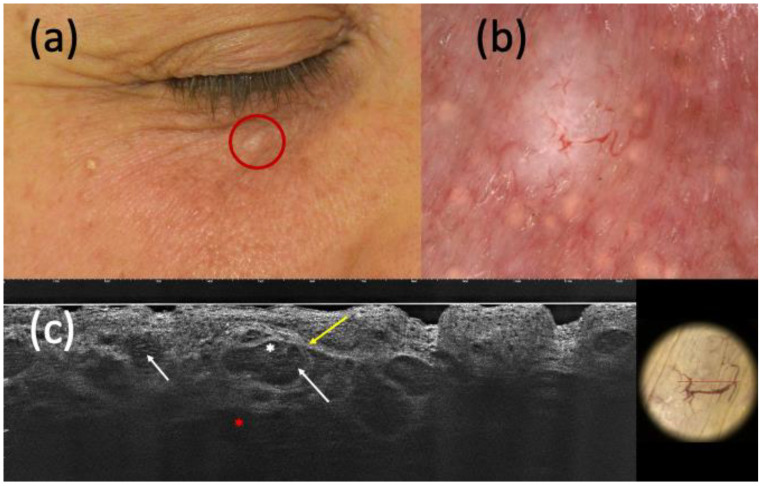
Nodular basal cell carcinoma (BCC) on histopathology, with non-specific clinical (**a**) and dermoscopic (**b**) examination but highly suspected in line-field confocal optical coherence tomography (LC-OCT) (**c**). It was a small whitish papule of the right inferior palpebra (**a**) that, under dermoscopy, displayed a homogeneous white pigmentation and branched vessels (**b**). The presence of hypo-reflective roundish lobules (white arrows), clefting (white asterisk), hyperreflective stroma (yellow arrow), and highly hypo-reflective areas corresponding to dilated vessels (red asterisk) in LC-OCT corroborated the hypothesis of BCC.

Considering the 152 completely excised and histologically confirmed BCCs, LC-OCT was able to identify 148 of them (Figure 1 and Figure 2), while dermoscopy identified only 141.

**Figure 3:**
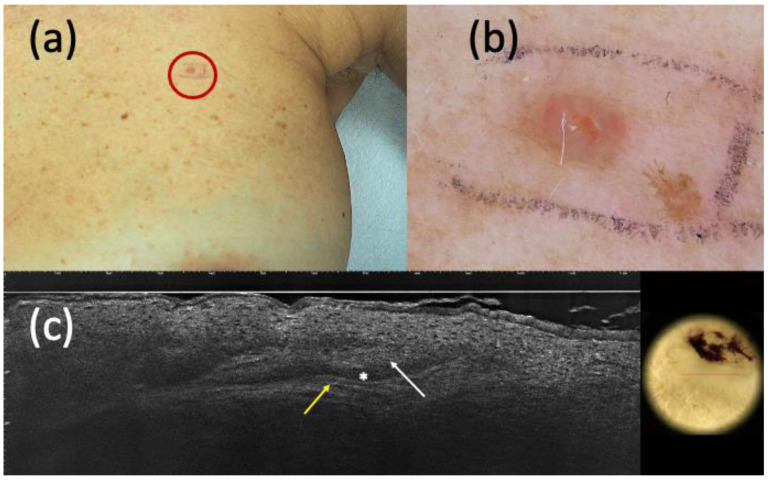
Suspected basal cell carcinoma (BCC) on clinical (**a**) and dermoscopic (**b**) examination; line-field confocal optical coherence tomography (LC-OCT) (**c**) supported the diagnosis of BCC. It was a pink papule of the left breast (**a**) that, under dermoscopy, appeared as a pinkish homogeneous pigmentation with central micro-ulceration (**b**). LC-OCT showed hypo-reflective roundish lobules (white arrow), clefting (white asterisk), and hyperreflective stroma (yellow arrow) (**c**). However, histopathology revealed the presence of an eccrine poroma.

**Figure 4:**
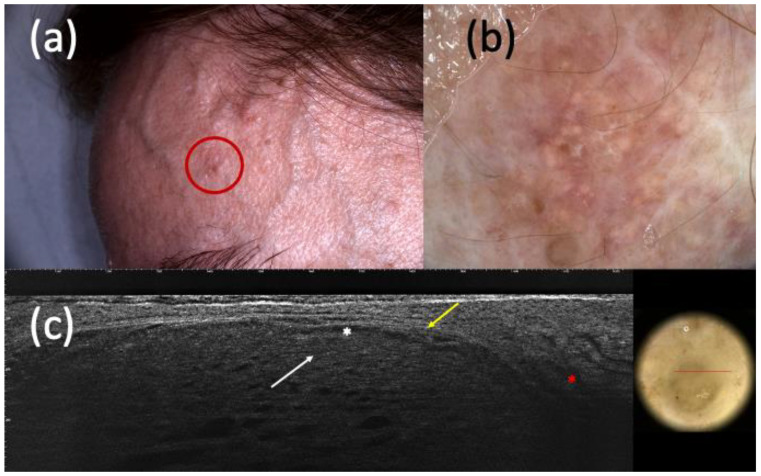
Suspected basal cell carcinoma (BCC) under clinical examination (**a**), dermoscopy (**b**), and line-field confocal optical coherence tomography (LC-OCT) (**c**). It was a dyschromic macule of the left frontotemporal region (**a**) that, in dermoscopy, showed shiny white streaks and short fine telangiectasias (**b**). In LC-OCT, hypo-reflective roundish lobules (white arrow), clefting (white asterisk), hyperreflective stroma (yellow arrow), and highly hypo-reflective areas corresponding to dilated vessels (red asterisk) were observed (**c**). However, the histologic examination revealed the existence of a trichoblastoma.

On the other hand, considering the 159 completely excised and histopathologically diagnosed non-BCC lesions, dermoscopy recognized 90 of them and LC-OCT 106. Moreover, dermoscopy was not able to identify 11 BCCs and misdiagnosed 31 BCCs, while LC-OCT exhibited a smaller margin of error by missing 4 BCCs and 17 BCC imitators (Figure 3 and Figure 4).

From these data, LC-OCT showed higher sensitivity (0.98, CI 0.95–1.00), specificity (0.94, CI 0.91–0.97), and accuracy (0.96, CI 0.94–0.98) than dermoscopy, which had a sensitivity of 0.93 (CI 0.88–0.96), a specificity of 0.56 (CI 0.49–0.62), and an accuracy of 0.73 (CI 0.69–0.77). In addition, LC-OCT had a PPV of 0.94 (CI 0.90–0.97) and an NPV of 0.98 (CI 0.96–1.00), while dermoscopy had a lower PPV (0.64, CI 0.59–0.70) and NPV (0.90, CI 0.83–0.94).

### 3.2. Second Endpoint: Malignant vs. Benign Considering Completely Excised and Histopathologically Confirmed Lesions

We examined the same group of lesions for the malignant (BCC, Bowen’s disease, SCC, and AK) vs. benign (IN, SK, SH, IL, and “other”) diagnosis. Of 208 malignant lesions completely excised and histologically diagnosed, 201 were correctly diagnosed as malignant by dermoscopy and 205 by LC-OCT. In comparison, 76 lesions were correctly identified as benign by dermoscopy and 86 by LC-OCT. A total of 7 malignant and 28 benign lesions at histology were misdiagnosed by dermoscopy. LC-OCT failed to recognize 3 malignant and 18 benign lesions. The sensitivity of dermoscopy was high (0.97, CI 0.93–0.99), but that of LC-OCT was higher (0.99, CI 0.96–1.00). The specificity was 0.73 (CI 0.64–0.81) for dermoscopy and 0.83 (CI 0.74–0.89) for LC-OCT. Accuracy was 0.89 (CI 0.85–0.92) for dermoscopy and 0.93 (CI 0.90–0.96) for LC-OCT. The PPV (0.92, CI 0.88–0.95) and NPV (0.97, CI 0.91–0.99) of LC-OCT were higher than dermoscopy (PPV of 0.88, CI 0.83–0.92 and NPV of 0.92, CI 0.83–0.97).

### 3.3. Third Endpoint: BCC vs. Imitators Considering All Histopathologically Confirmed and Follow-Up Lesions

Analyzing all the histopathologically diagnosed and the follow-up lesions (total number of 466), we observed that LC-OCT diagnosed 212 lesions as BCCs and 236 as BCC imitators, while it could not identify 4 BCCs and 14 non-BCCs. Nonetheless, dermoscopy could only recognize 200 BCCs and 139 imitators and missed the identification of 16 BCCs and 111 non-BCCs. It appeared that the specificity of LC-OCT was far higher (0.94, CI 0.91–0.97) than that of dermoscopy (0.56, CI 0.49–0.62); the sensitivity was 0.98 (CI 0.95–1.00) for LC-OCT and 0.93 (CI 0.88–0.96) for dermoscopy, and accuracy came out as 0.96 (CI 0.94–0.98) for LC-OCT and 0.73 (CI 0.69–0.77) for dermoscopy. Similar to the first endpoint, LC-OCT showed a PPV of 0.94 (CI 0.90–0.97) and an NPV of 0.98 (CI 0.96–1.00), while dermoscopy had a PPV of 0.64 (CI, 0.59–0.70) and an NPV of 0.90 (CI 0.84–0.94).

### 3.4. Fourth Endpoint: Malignant vs. Benign Considering All Histopathologically Confirmed and Follow-Up Lesions

The last sub-analysis was performed considering both histopathologically confirmed and follow-up lesions and the diagnostic ability of LC-OCT and dermoscopy in differentiating the most frequent NMSCs (BCC, Bowen’s disease, and SCC), including AK, from benign lesions. The overall performances of dermoscopy and LC-OCT showed that the optical imaging device correctly identified 288 malignant and 157 benign lesions but missed 18 benign and 3 malignant ones. Dermoscopy only diagnosed 282 malignant and 76 benign lesions and failed to identify 99 benign and 9 malignant ones. Also in that case, the specificity was higher for LC-OCT (0.90, CI 0.84–0.94) than for dermoscopy (0.43, CI 0.36–0.51); the sensitivity was 0.99 (CI 0.97–1.00) for LC-OCT and 0.97 (CI 0.94–0.99) for dermoscopy, and accuracy appeared as 0.96 (CI 0.93–0.97) in LC-OCT and 0.77 (CI 0.73–0.81) in dermoscopy. PPV was 0.94 (CI 0.91–0.97) for LC-OCT and 0.74 (CI 0.69–0.78) for dermoscopy, and NPV was 0.98 (CI 0.95–1.00) for LC-OCT and 0.89 (CI 0.81–0.95) for dermoscopy.

## 4. Discussion

LC-OCT had a higher diagnostic ability than dermoscopy in differentiating BCCs from its imitators and skin carcinomas from benign skin lesions. We found a higher sensitivity, specificity, and diagnostic accuracy of LC-OCT than dermoscopy, both considering completely excised lesions alone and the full analysis with biopsied and follow-up cases. The latter analysis, performed on a larger series (466 vs. 312 cases), enhanced the higher diagnostic ability of LC-OCT than dermoscopy.

Regarding the ability to distinguish BCC from its imitators, LC-OCT supported the hypothesis of BCC when suspected by clinical examination and/or dermoscopy in most cases. Considering both histopathologically confirmed and follow-up lesions, the sensitivity of LC-OCT was 0.98 (CI 0.95–1.00), and that of dermoscopy was 0.93 (CI 0.88–0.96). LC-OCT highly increased diagnostic confidence by highlighting the presence of BCC when not highly clinically suspected, as shown in Figure 1 and Figure 2. Moreover, this new non-invasive imaging technique helped the exclusion of the presence of BCCs. Dermoscopy had 111 false positive BCCs, and LC-OCT identified 85 non-BCC lesions (confirmed by histopathology after being biopsied) among these cases suspected of being BCCs based on dermoscopic examination. These data explained the higher specificity of LC-OCT than dermoscopy, especially when we considered the analysis of all the excised, biopsied, and follow-up cases (specificity of 0.94, CI 0.91–0.97 for LC-OCT vs. specificity of 0.56, CI 0.49–0.62 for dermoscopy). Although all of these 85 skin lesions were biopsied according to our conventional approach, their biopsies could have been spared by relying on LC-OCT examination. There were few false-positive cases diagnosed as BCCs by LC-OCT that corresponded to three Bowen’s diseases, an SK, an SH, three ILs, an eccrine poroma (Figure 3), a trichoblastoma (Figure 4), a sebaceoma, a trichilemmoma, and two cases of healthy skin. These data revealed the superiority of NPV (0,97, CI 0.91–0.99 in LC-OCT and 0.92, CI 0.83–0.97 in dermoscopy) over PPV (0.92, CI 0.88–0.95 in LC-OCT and 0.88, CI 0.83–0.92 in dermoscopy) for both diagnostic tools. LC-OCT was better at ruling out the presence of a BCC than at specifically diagnosing it, and its performance was superior to dermoscopy.

Concerning the diagnosis of skin malignancy (i.e., 216 BCCs, 14 Bowen’s diseases, 22 SCCs, and 39 AKs) vs. benignity (IN, SK, SH, IL, and “other”), similar results were obtained. Considering all histopathologically confirmed and follow-up lesions, LC-OCT showed higher sensitivity (0.99, CI 0.97–1.00) and higher specificity (0.90, CI 0.84–0.94) than dermoscopy, which presented 0.97 (CI 0.94–0.99) for sensitivity and 0.43 (CI 0.36–0.51) for specificity. The data were mainly driven by the high number of BCCs (216 histologically confirmed), which are the most common skin carcinomas, as demonstrated by our series. Interestingly, LC-OCT identified as benign five lesions that were suspicious for being SCCs at dermoscopy. These data revealed a higher specificity of LC-OCT when distinguishing NMSCs from benign lesions. A higher NPV (0.98, CI 0.95–1.00) of LC-OCT than dermoscopy (0.89, CI 0.81–0.95) confirmed the previous results. The improved ability to identify malignant lesions (true positive cases) with LC-OCT was demonstrated by the difference between the PPV of LC-OCT (0.94, CI 0.91–0.97) and that of dermoscopy (0.74, CI 0.69–0.78).

LC-OCT is a second-level examination performed on lesions selected by clinical and dermoscopic examination. It is not used as a screening tool due to its longer examination time (a few minutes) compared to dermoscopy (a few seconds). In LC-OCT, single keratinocytes inside the epidermis are well visible and can be identified by the presence of their nuclei, which are seen as central roundish hypo-reflective areas with the surrounding medium-reflective cytoplasm. In normal skin, the peripheral contours of keratinocytes are visible on horizontal sections and not on vertical sections. In the case of AK and SCC, their irregularity in shape and size is mainly perceived by the irregularity of their nuclei [24]. However, single cells of BCC are not always visible because they can be crowded and form the so-called “millefeuille pattern” that corresponds to the dense cellularity within the basaloid tumor island, composed of basaloid cells, immune cells, apoptotic bodies, and mitotic figures. The pattern appears like horizontal lines determined by the medium reflection of the cytoplasm of the cells (gray laminated structures) separated by dark holes corresponding to cell nuclei [20]. Although peripheral palisading of BCC is less visible than in histopathology and RCM and cannot be selected as a distinctive LC-OCT criterion for BCC diagnosis, with LC-OCT, we can appreciate cells piled one on the other at the periphery of the tumor islands with flat dark overlapped nuclei that have the same orientation. The millefeuille pattern is relevant to differentiate BCC from other entities characterized by dermal lobules, such as SH (characteristic lobules with granular-lobular pattern), IN (characteristic lobules with wave-like pattern), melanoma (characteristic lobules with atypical melanocytic cells), and cutaneous vascular lesions (characteristic lobules with dark vascular lacunae) [26].

The majority of the cases of our study were included in a similar work of our group that evaluated the diagnostic accuracy of LC-OCT for the diagnosis of skin carcinomas in one year of clinical activity [19]. The study mainly focused on BCCs; only 22 histologically diagnosed SCCs or Bowen’s diseases were analyzed. Considering both histopathologically diagnosed and follow-up cases and the ability to identify skin cancers, the authors reported a significant increase in specificity, from 0.64 (CI 0.55–0.72) for dermoscopy to 0.89 (CI 0.82–0.93) for LC-OCT. The sensitivity was similar for the two non-invasive techniques (0.95, CI 0.89–0.98 for dermoscopy and 0.95, CI 0.90–0.99 for LC-OCT). These data were predominantly determined by the 79 histologically confirmed BCCs and were similar to those of the present study. The sensitivity values were the same, while the specificity increased in the current study. This trend is possibly explained by an increasing learning curve in the interpretation of LC-OCT images thanks to a longer period of use of the device. In our experience, the diagnosis of BCC seems to be quite easy under LC-OCT due to the peculiar aspect of the tumor islands characterized by hypo-reflective lobules with internal millefeuille patterns surrounded by clefting and hyperreflective stroma [20] that can be recognized in a few weeks of activity, whereas SCC and Bowen’s disease require longer expertise with the ability to identify atypical keratinocytes and the interruption of the dermal–epidermal junction [24,25]. The LC-OCT device is provided by a dermatoscopic probe that facilitates the identification of a lesion to be examined, giving a real-time correspondence between dermoscopy and LC-OCT images. An Artificial Intelligence (AI)--based prototype software was developed to identify BCC lobules in LC-OCT. It is reasonable that this tool can improve the LC-OCT diagnosis of BCC and that a similar one can be developed for the identification of SCC and Bowen’s disease.

The main limitation of the two studies of our group was that all diagnoses were performed by a single skin imaging expert (E.C.). Moreover, dermoscopic and LC-OCT diagnoses were based on the investigator’s experience and not on precisely defined diagnostic criteria. Defining diagnostic algorithms for the future LC-OCT diagnosis of skin tumors could provide more reliable and comparable results. NMSCs different from BCC, Bowen’s disease, and SCC were excluded due to the lack of established LC-OCT diagnostic criteria. Only a few reports about LC-OCT findings of rarer NMSCs are available [5,7,8]. There was a low number of Bowen’s diseases and SCCs in comparison with BCCs because their prevalence is lower, their acquisition with LC-OCT is not always possible due to hyperkeratosis, and their LC-OCT diagnostic criteria are less defined [24,25]. Melanocytic lesions different from IN were also excluded because of the lack of LC-OCT criteria. The dermoscopic criteria of melanocytic lesions greatly improved the clinical examination, but to date, the literature about the LC-OCT of those entities is scarce [4,26].

To complete our study, we performed an additional analysis, including AKs in the group of benign lesions since they do not require surgical management. The endpoints were the same. Considering all the histopathologically confirmed and follow-up lesions, the ability of LC-OCT to differentiate malignant from benign (including AK) lesions had a sensitivity of 0.97 (CI 0.94–0.99), specificity of 0.90 (CI 0.85–0.94), and accuracy of 0.91 (CI 0.91–0.96). Dermoscopy showed lower performances with a sensitivity of 0.93 (CI 0.89–0.96), specificity of 0.43 (CI 0.36–0.49), and accuracy of 0.70 (CI 0.66–0.74). Although specificity remained unaltered, both sensitivity and diagnostic accuracy were lower when considering AK as a benign lesion with 0.97 vs. 0.99 (sensitivity) and 0.94 vs. 0.96 (accuracy) for LC-OCT and 0.93 vs. 0.97 (sensitivity) and 0.70 vs. 0.77 (accuracy) for dermoscopy.

Our patient cohort was preselected since every patient with a lesion suspicious of skin cancer was referred by a dermatologist. The eyelid margin, internal cantus of the eye, and upper eyelid were excluded from this study. In the literature, we could find only another prospective real-life study on the diagnostic accuracy of LC-OCT for skin tumors [25]. The study observed a significantly increased diagnostic accuracy of LC-OCT compared to dermoscopy. Nevertheless, while the sensitivity of LC-OCT (0.98) was higher than dermoscopy (0.90), the specificity of LC-OCT (0.80) compared to dermoscopy (0.86) was lower. The authors also analyzed the diagnoses from the point of view of confidence in making them and found that 70% of LC-OCT diagnoses were provided with high confidence compared to 48% of high-confidence dermoscopic diagnoses. In that case, the results significantly increased the LC-OCT performances (100% of sensitivity and 97% of specificity).

## 5. Conclusions

In conclusion, we could assert that LC-OCT had a higher specificity than dermoscopy for the differential diagnosis between BCC and its imitators but also between the most frequent NMSCs (i.e., BCC, Bowen’s disease, and SCC) and benign lesions. LC-OCT was better at excluding malignity than giving a precise diagnosis. The diagnostic accuracy was also higher for LC-OCT than for dermoscopy, especially when considering all types of NMSCs together, while the sensitivity only slightly increased in LC-OCT. Further studies with different databases are needed to confirm our findings.

## Figures and Tables

**Table 1:** Confusion matrix of the 360 completely excised and histologically confirmed lesions: dermoscopy vs. histology (a.) and line-field confocal optical coherence tomography (LC-OCT) vs. histology (b.).

**a**		**HISTOLOGY**													
	**DIAGNOSIS**	**BCC**	**AK**	**BD**	**SCC**	**BQ**	**ID**	**KS**	**SH**	**IL**	**O**	**L**	**N**	**M**	**TOTAL**
**DERMOSCOPY**	**BCC**	141	0	5	0	5	1	1	1	3	15	2	1	0	**175**
	**AK**	3	14	1	1	1	0	0	0	0	1	0	0	0	**21**
	**BD**	0	1	4	1	1	0	0	0	1	0	0	0	0	**8**
	**SCC**	4	4	3	18	0	0	1	0	0	4	0	0	0	**34**
	**BQ**	0	0	0	0	0	0	0	0	0	0	0	0	0	**0**
	**ID**	0	0	0	0	0	1	0	0	0	0	0	1	0	**2**
	**KS**	2	0	1	1	0	0	8	0	0	5	0	0	0	**17**
	**SH**	0	0	0	0	0	0	0	0	0	1	0	0	0	**1**
	**IL**	0	0	0	0	0	0	0	0	4	3	0	0	0	**7**
	**O**	2	0	0	1	0	0	3	0	7	41	0	0	0	**54**
	**L**	0	0	0	0	0	0	0	0	0	0	1	0	0	**1**
	**N**	0	0	0	0	0	2	1	0	0	0	0	18	1	**22**
	**M**	0	1	0	0	0	0	0	0	0	0	0	3	14	**18**
	**TOTAL**	**152**	**20**	**14**	**22**	**7**	**4**	**14**	**1**	**15**	**70**	**3**	**23**	**15**	**360**
**b**		**HISTOLOGY**													
	**DIAGNOSIS**	**BCC**	**AK**	**BD**	**SCC**	**BQ**	**ID**	**KS**	**SH**	**IL**	**O**	**L**	**N**	**M**	**TOTAL**
**LC-OCT**	**BCC**	148	0	3	0	3	0	1	1	3	6	1	0	0	**166**
	**AK**	1	16	2	1	0	0	0	0	0	1	0	0	0	**21**
	**BD**	0	1	5	0	2	0	0	0	1	0	0	0	0	**9**
	**SCC**	2	2	4	19	0	0	1	0	0	3	0	0	0	**31**
	**BQ**	0	0	0	0	1	0	0	0	0	0	0	0	0	**1**
	**ID**	0	0	0	0	0	1	0	0	0	0	0	1	0	**2**
	**KS**	0	0	0	1	0	0	10	0	0	4	0	0	1	**16**
	**SH**	0	0	0	0	0	0	0	0	0	1	0	0	0	**1**
	**IL**	0	0	0	0	0	0	0	0	5	3	0	0	0	**8**
	**O**	1	0	0	1	1	0	2	0	6	49	1	0	1	**62**
	**L**	0	0	0	0	0	0	0	0	0	1	1	0	0	**2**
	**N**	0	0	0	0	0	3	0	0	0	1	0	20	1	**25**
	**M**	0	1	0	0	0	0	0	0	0	1	0	2	12	**16**
	**TOTAL**	**152**	**20**	**14**	**22**	**7**	**4**	**14**	**1**	**15**	**70**	**3**	**23**	**15**	**360**

BCC, basal cell carcinoma; AK, actinic keratosis; BD, Bowen’s disease; SCC, squamous cell carcinoma; ID, intradermal nevus; KS seborrheic keratosis; SH, sebaceous hyperplasia; IL, inflammatory lesion; O, other; L, lentigo; N, nevus; and M, melanoma.

**Table 2:** Confusion matrix of the 466 histopathologically diagnosed or followed-up lesions: dermoscopy vs. histology and follow-up (a.) and line-field confocal optical coherence tomography (LC-OCT) vs. histology and follow-up (b.).

**a**		**HISTOLOGY and FOLLOW-UP**									
	**DIAGNOSIS**	**BCC**	**AK**	**BD**	**SCC**	**ID**	**KS**	**SH**	**IL**	**O**	**TOTAL**
**DERMOSCOPY**	**BCC**	200	17	5	0	7	3	5	25	49	**311**
	**AK**	6	14	1	1	0	0	0	0	1	**23**
	**BD**	0	1	4	1	0	0	0	1	0	**7**
	**SCC**	4	6	3	18	0	2	0	1	5	**39**
	**ID**	0	0	0	0	1	0	0	0	0	**1**
	**KS**	3	0	1	1	0	8	0	0	5	**18**
	**SH**	0	0	0	0	0	0	0	0	1	**1**
	**IL**	0	0	0	0	0	0	0	4	3	**7**
	**O**	2	0	0	1	0	3	0	7	41	**54**
	**L**	0	0	0	0	0	0	0	0	0	**0**
	**N**	1	0	0	0	2	1	0	0	0	**4**
	**M**	0	1	0	0	0	0	0	0	0	**1**
	**TOTAL**	**216**	**39**	**14**	**22**	**10**	**17**	**5**	**38**	**105**	**466**
**b**		**HISTOLOGY and FOLLOW-UP**									
	**DIAGNOSIS**	**BCC**	**AK**	**BD**	**SCC**	**ID**	**KS**	**SH**	**IL**	**O**	**TOTAL**
**LC-OCT**	**BCC**	212	0	3	0	0	1	1	3	6	**226**
	**AK**	1	35	2	1	0	0	0	0	1	**40**
	**BD**	0	1	5	0	0	0	0	1	0	**7**
	**SCC**	2	2	4	19	0	1	0	0	3	**31**
	**ID**	0	0	0	0	7	0	0	0	0	**7**
	**KS**	0	0	0	1	0	13	0	0	4	**18**
	**SH**	0	0	0	0	0	0	4	0	1	**5**
	**IL**	0	0	0	0	0	0	0	28	3	**31**
	**O**	1	0	0	1	0	2	0	6	84	**94**
	**L**	0	0	0	0	0	0	0	0	1	**1**
	**N**	0	0	0	0	3	0	0	0	1	**4**
	**M**	0	1	0	0	0	0	0	0	1	**2**
	**TOTAL**	**216**	**39**	**14**	**22**	**10**	**17**	**5**	**38**	**105**	**466**

BCC, basal cell carcinoma; AK, actinic keratosis; BD, Bowen’s disease; SCC, squamous cell carcinoma; ID, intradermal nevus; KS seborrheic keratosis; SH, sebaceous hyperplasia; IL, inflammatory lesion; O, other; L, lentigo; N, nevus; and M, melanoma.

## Data Availability

The data presented in this study are available on request from the corresponding author.
